# Three-dimensional echocardiography for the assessment of left ventricular geometry and papillary muscle morphology in hypertrophic cardiomyopathy

**DOI:** 10.1007/s40477-017-0277-y

**Published:** 2018-01-06

**Authors:** Mustafa Erden, Hannah G. van Velzen, Myrthe E. Menting, Annemien E. van den Bosch, Ben Ren, Michelle Michels, Wim B. Vletter, Ron T. van Domburg, Arend F.L. Schinkel

**Affiliations:** 1 Department of Cardiology, Thoraxcenter, Erasmus Medical Center, Rotterdam, The Netherlands; 2 Department of Cardiology, Erasmus MC, Thoraxcenter Room Ba304, ‘s-Gravendijkwal 230, 3015 Rotterdam, The Netherlands

**Keywords:** Hypertrophic cardiomyopathy, 3D echocardiography, Papillary muscle

## Abstract

**Background:**

Hypertrophic cardiomyopathy (HC) is characterized by left ventricular (LV) hypertrophy and associated with papillary muscle (PM) abnormalities. The aim of this study was to evaluate the utility of three-dimensional echocardiography (3DE) for the geometric assessment of LV hypertrophy and PM morphology.

**Methods:**

The study included 24 patients with an established diagnosis of HC and 31 healthy controls. 3DE was performed using an iE33 or EPIQ 7C ultrasound system with an X5-1 transducer. QLAB software was used for the 3D analysis of LV wall thickness (LVWT) and PM morphology and hypertrophy; the number and cross-sectional area (CSA) of anterolateral and posteromedial PMs; and the presence of bifid or accessory PMs.

**Results:**

Patients with HC had a larger LVWT compared to controls in all segments (*p* < 0.001), and LVWT was largest in the midventricular septal segment (2.12 ± 0.68 cm). The maximum LVWT followed a spiral pattern from the LV base to the apex. The CSA of both anterolateral and posteromedial PMs was larger in patients with HC than in controls (1.92 vs. 1.15 cm^2^; *p* = 0.001 and 1.46 vs. 1.08 cm^2^; *p* = 0.033, respectively). The CSA of the posteromedial PM was larger in patients with LVOT obstruction than in those without (2.64 vs 1.16 cm^2^, *p* = 0.021).

**Conclusions:**

3DE allows the assessment of LV geometry and PM abnormalities in patients with HC. 3DE demonstrated that the maximum hypertrophy was variable and generally located in a spiral from the LV base to the apex.

## Sommario

**Premessa:**

La cardiomiopatia ipertrofica (HC) è caratterizzata da ipertrofia del ventricolo sinistro (LV) e associata ad anomalie del muscolo papillare (PM).

**Scopo dello studio:**

Lo scopo di questo lo studio è stato quello di valutare l’utilità dell’ecocardiografia tridimensionale (3DE) nell’ottenere una valutazione geometrica dell’ipertrofia del VS e della morfologia del PM.

**Metodi:**

Lo studio ha incluso 24 pazienti con diagnosi accertata di HC e 31 controlli sani. La 3DE è stata eseguita utilizzando un sistema ecografico iE33 o EPIQ 7C con un trasduttore X5-1. Il software QLAB è stato utilizzato per l’analisi 3D dello spessore della parete del LV (LVWT) e della morfologia e ipertrofia del PM; il numero e l’area della sezione trasversale (CSA) del PM anterolaterale e posteromediale e definire la presenza di PM bifidi o accessori.

**Risultati:**

I pazienti con HC avevano una LVWT maggiore rispetto ai controlli in tutti i segmenti (*p* < 0,001) e LVWT era maggiore nel segmento del setto medio-ventricolare (2,12 ± 0,68 cm). La LVWT massima seguiva uno schema a spirale dalla base LV all’apice. La CSA di entrambi i PM anterolaterali e posteromediali era maggiore nei pazienti con HC rispetto ai controlli (1,92 vs 1,15 cm^2^; *p* = 0,001 e 1,46 vs 1,08 cm^2^; *p* = 0,033 rispettivamente). La CSA del PM posteromediale era maggiore nei pazienti con ostruzione LVOT rispetto a quelli senza (2,64 vs 1,16 cm^2^, *p* = 0,021).

**Conclusioni:**

La 3DE consente di valutare la geometria LV e le anomalie di PM in pazienti con HC. La 3DE ha dimostrato che l’ipertrofia massima era variabile e generalmente conformata a spirale dalla base del LV all’apice.

## Introduction

Hypertrophic cardiomyopathy (HC) is the most common inherited cardiac disease, with an estimated prevalence of 1 in 500 [1]. Hypertrophic cardiomyopathy is characterized by a broad clinical and morphological spectrum including left ventricular (LV) hypertrophy and abnormal LV papillary muscle (PM) morphology and thickness [2–7]. Currently, two-dimensional echocardiography (2DE) is the most frequently used imaging modality for the diagnosis and follow-up of patients with HC [8, 9]. Clearly, 2DE has inherent limitations that may be overcome by three-dimensional echocardiography (3DE). There are indications that 3DE allows a better geometric assessment of PM morphology and LV hypertrophy and may provide information that alters clinical decision making [9, 10]. The aim of this study was to assess the utility of 3DE for the assessment of LV geometry and PM abnormalities in patients with HC as compared to healthy controls. Additionally, the relation between PM abnormalities and left ventricular outflow tract (LVOT) obstruction was studied.

## Methods

### Patient population and study protocol

This study included 24 consecutive patients with an established diagnosis of HC and 31 age-matched healthy controls. All patients underwent standard 2DE in conjunction with 3DE. Only patients with sufficient image quality were included in this study. The diagnosis of HC was based on a LV wall thickness (LVWT) ≥ 15 mm that was not explained by loading conditions. LVOT obstruction was defined as a resting or provocable gradient ≥ 30 mmHg assessed by Doppler echocardiography. Patients with HC linked to Noonan’s syndrome, Fabry’s disease, or congenital heart defects were excluded.

### Echocardiography

3DE was performed using an iE33 or EPIQ 7C ultrasound system (Philips, Best, The Netherlands) with a X5-1t matrix-array transducer. Electrocardiographically gated full-volume datasets of the LV (built from four subvolumes) were acquired in the parasternal long axis and apical views during breath-hold. Care was taken to include the complete LV including the PM, and septum within the imaging volume throughout the acquisition by adjusting the lateral and elevation widths of the acquisition sector. Each full-volume dataset was digitally stored and exported to QLAB 3DQA software (Philips, Best, The Netherlands) for offline analysis.

The full-volume LV 3DE dataset was displayed as three orthogonal multiplanar reconstruction views. For the analysis of end-diastolic LVWT, a 16-segment model was used, according to EAE/ASE recommendations [11]. The short-axis end-diastolic frames that provided the best visualization of the endocardial and epicardial borders were selected. The LVWT was assessed at the center of each myocardial segment from the leading endocardial edge to the leading epicardial edge. The geometric pattern of LV hypertrophy was determined by the location and extent of hypertrophy at basal, midventricular and apical level. For the PM analysis, the anterolateral PM (ALPM) and posteromedial PM (PPM) were identified. Subsequently, morphology of the PMs was assessed: Individual PMs were assessed using long- and short-axis images on different levels to identify bifid, double bifid and accessory PMs. The cross-sectional area (CSA) of each PM was measured in the short-axis view at midventricular level, and total CSA was calculated for the ALPM and PPM.

### Statistical analyses

Statistical analyses were performed using SPSS 21 (IBM, Armonk, New York). Continuous variables are reported as mean ± standard deviation. Categorical variables are expressed as number (%). Mann–Whitney *U* test was used to compare the continuous variables and the Chi-square test was used to compare categorical variables. A *p* value < 0.05 was considered statistically significant.

## Results

### Patient characteristics

The clinical characteristics of patients with HC and controls are summarized in Table 1. The age of the patients with HC and controls was comparable (35 ± 14 vs 33 ± 7 years, *p* = 0.173), and the patient group included significantly more men (88% vs 42%, *p* < 0.05).

**Table 1:** Characteristics of patients with hypertrophic cardiomyopathy (HC) and healthy controls

Variable	Patients with HC (*n* = 24)	Controls (*n* = 31)	*p* value
Age (years)	35 ± 14	33 ± 7	0.173
Men	21 (88%)	13 (42%)	< 0.05
BMI (kg/m^2^)	23 ± 5	23 ± 2	
BSA (m^2^)	1.83 ± 0.32	1.85 ± 0.18	
Angina	3 (13%)	0	
Dyspnea	6 (25%)	0	
NYHA class ≥ II	6 (25%)	0	
Palpitations	6 (25%)	0	
LVOT obstruction (≥ 30 mmHg)	6 (25%)	0	
Pathogenic mutations			
Myosin-binding protein C	8 (33%)	–	
Myosin heavy chain	2 (8%)	–	
Troponin I	1 (4%)	–	
Mitochondrial DNA	1 (4%)	–	

Data are presented as mean ± standard deviation or number (%)

*BSA* body surface area, *LVOT* left ventricular outflow tract, *NYHA* New York Heart Association functional class

### Echocardiographic findings

Measurements of LVWT obtained from 3DE are presented in Table 2. Overall, LVWT in all 16 segments was larger in patients with HC than in controls (*p* < 0.001). The maximal LVWT in patients with HC at basal LV level was observed in the inferoseptal segments (1.87 ± 0.62 cm) and anteroseptal segments (1.87 ± 0.38 cm); at midventricular level in the anteroseptal segments (2.18 ± 0.67 cm); and at apical level in the lateral segment (1.76 ± 0.55 cm). The segments with maximal hypertrophy formed a spiral pattern from the base to the apex of the LV.

**Table 2:** Left ventricular wall thickness (LVWT) assessed by three-dimensional echocardiography (3DE)

Variable	Patients with HC (*n* = 24)	Controls (*n* = 31)	*p* value
Basal level			
Anterior	1.43 ± 0.37	0.84 ± 0.12	< 0.001
Anteroseptal	1.87 ± 0.38	0.86 ± 0.21	< 0.001
Inferoseptal	1.87 ± 0.62	0.85 ± 0.16	< 0.001
Inferior	1.41 ± 0.49	0.80 ± 0.15	< 0.001
Inferolateral	1.25 ± 0.26	0.76 ± 0.15	< 0.001
Anterolateral	1.25 ± 0.31	0.73 ± 0.14	< 0.001
Midventricular level			
Anterior	1.54 ± 0.36	0.86 ± 0.17	< 0.001
Anteroseptal	2.18 ± 0.67	0.87 ± 0.20	< 0.001
Inferoseptal	2.17 ± 0.73	0.89 ± 0.14	< 0.001
Inferior	1.47 ± 0.42	0.85 ± 0.16	< 0.001
Inferolateral	1.53 ± 0.51	0.75 ± 0.14	< 0.001
Anterolateral	1.46 ± 0.46	0.73 ± 0.15	< 0.001
Apical level			
Anterior	1.52 ± 0.28	1.03 ± 0.43	< 0.001
Septal	1.69 ± 0.71	1.01 ± 0.23	< 0.001
Inferior	1.49 ± 0.45	0.85 ± 0.21	< 0.001
Lateral	1.76 ± 0.55	0.94 ± 0.35	< 0.001

Data are presented as mean ± standard deviation in centimeter

*HC* hypertrophic cardiomyopathy

**Fig. 1:**
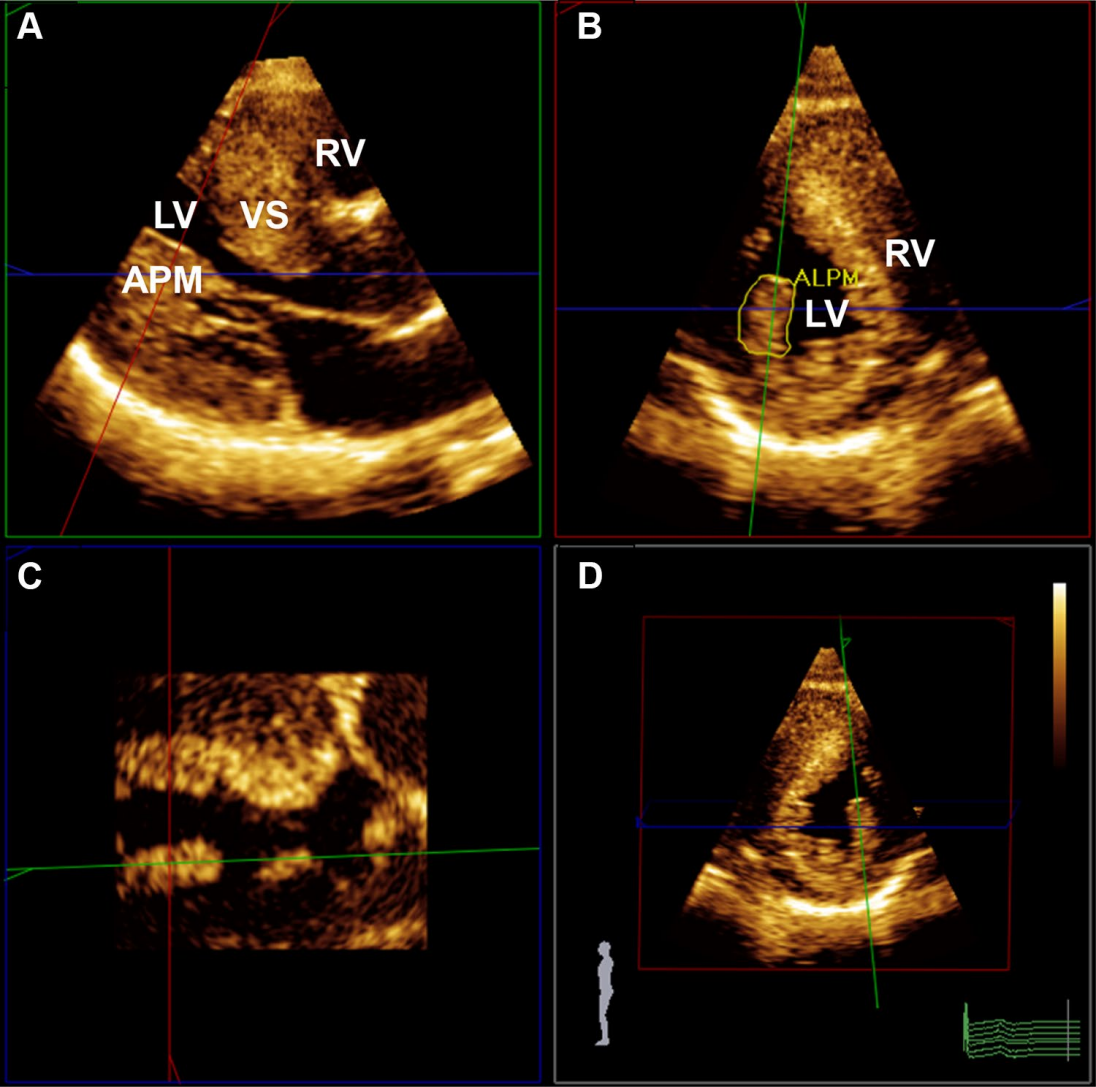
3DE assessment of anterolateral papillary muscle (ALPM) cross-sectional area in a patient with HC. **a** Long-axis plane; **b** Short-axis plane; **c** Coronal plane; **d** Alternative real time 3D. *ALPM* anterolateral papillary muscle, *LV* left ventricle, *RV* right ventricle, *VS* ventricular septum

**Fig. 2:**
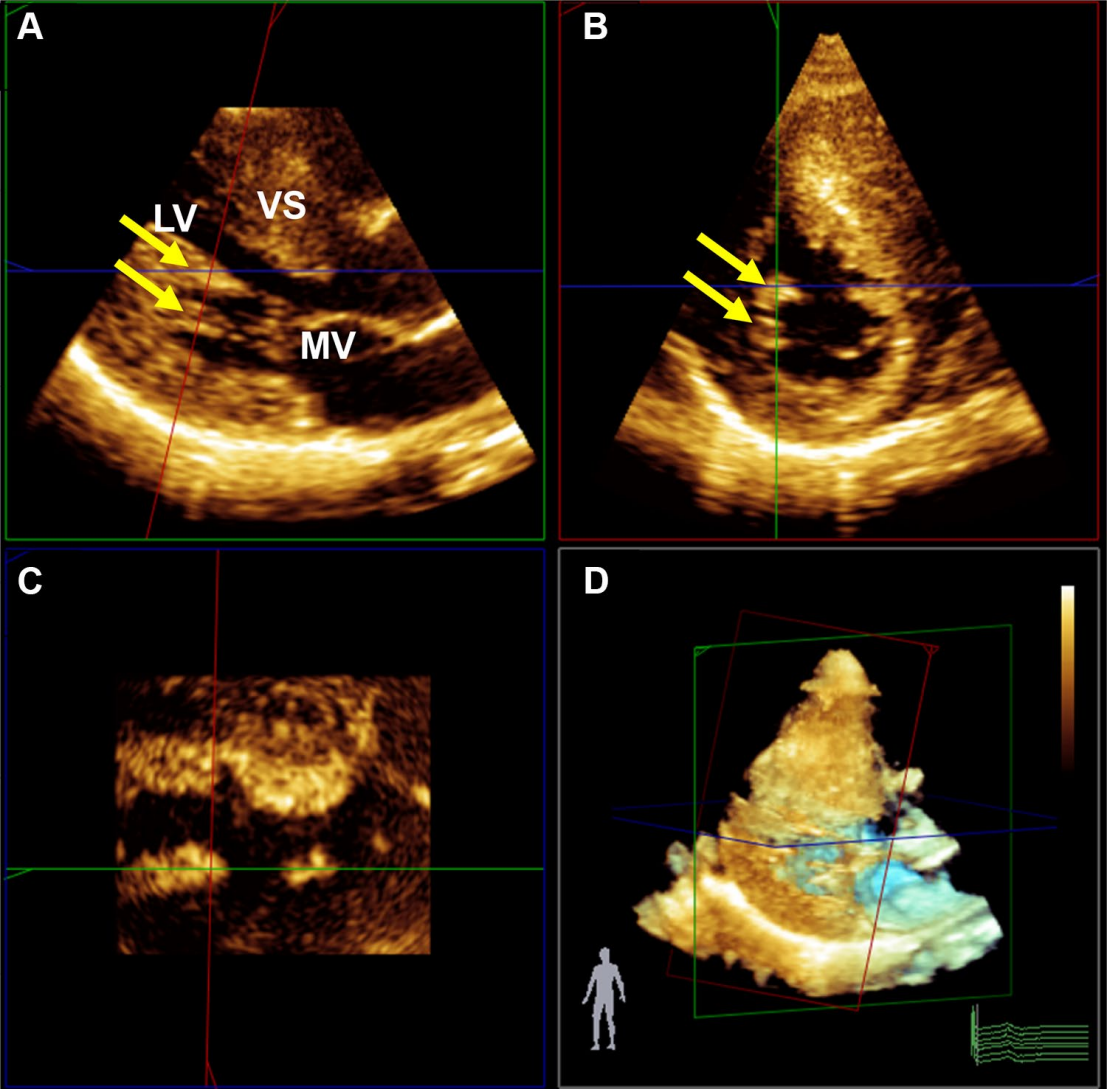
3DE assessment of a bifid PM in a patient with HC (yellow arrows). **a** Long-axis plane, **b** Short-axis plane; **c** Coronal plane; **d** Real time 3D. *LV* left ventricle, *MV* mitral valve, *VS* ventricular septum

**Fig. 3:**
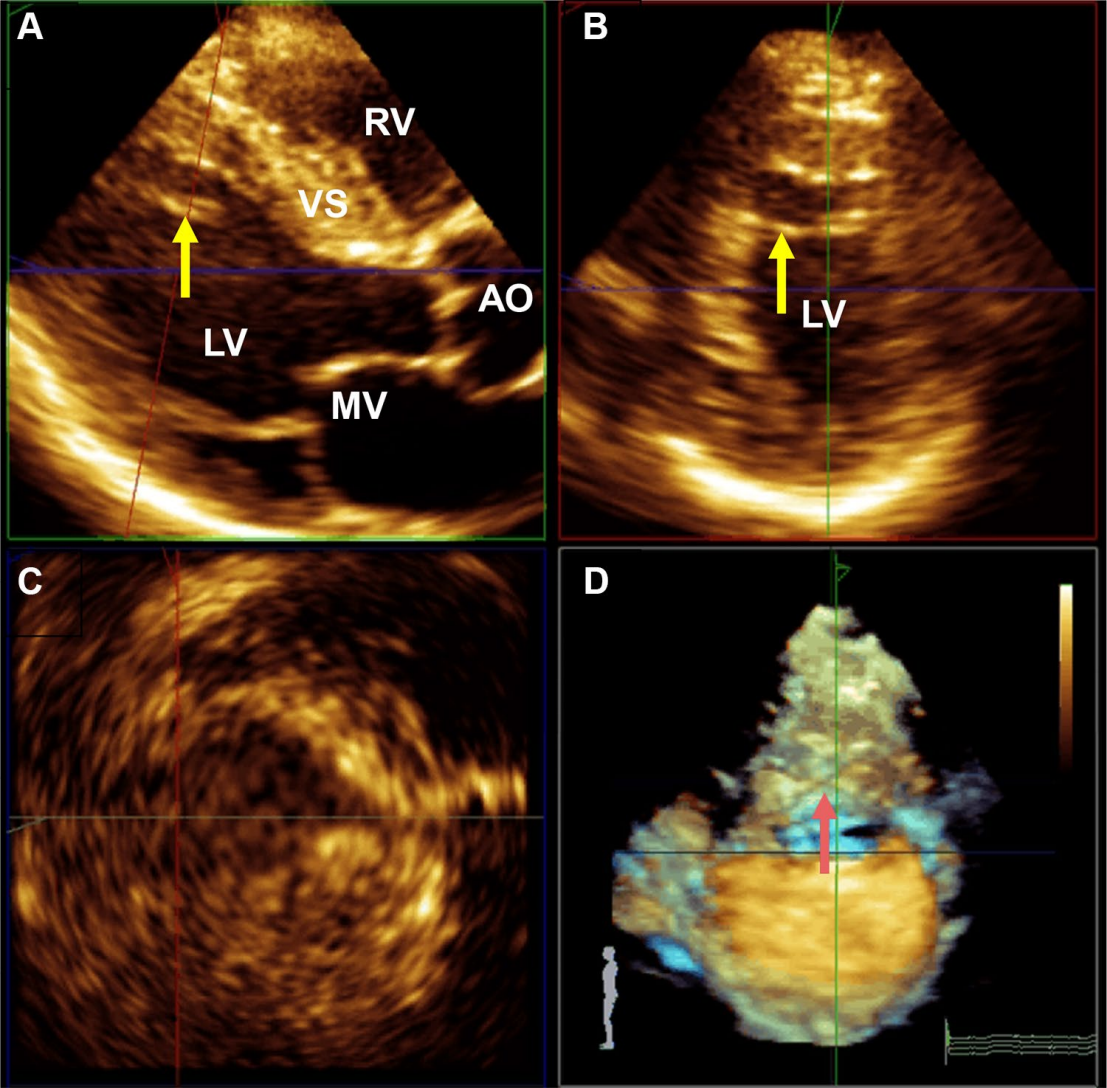
3DE assessment of an accessory papillary muscle in a patient with HC (yellow and red arrows). **a** Long-axis plane; **b** Short-axis plane; **c** Coronal Plane; **d** Real time 3D. *Ao* ascending aorta, *LV* left ventricle, *MV* mitral valve, *RV* right ventricle, *VS* ventricular septum

Measurements of PMs obtained from 3DE are presented in Table 3. The total ALPM and total PPM CSA were significantly larger in patients with HC than in controls (1.92 ± 0.81 vs. 1.15 ± 0.47 cm^2^; *p* = 0.001 and 1.46 ± 0.62 vs. 1.08 ± 0.37 cm^2^; *p* = 0.031, respectively). Figure 1 demonstrates an example of the 3DE analysis of the PM area. There was no significant difference in the number of ALPMs and PPMs between patients with HC and controls. Moreover, bifid and accessory PMs were not observed more frequently in patients with HC than in controls. Figure 2 shows an example of a patient with HC and a bifid PM, and Fig. 3 demonstrates an example of an accessory PM visualized by 3DE.

**Table 3:** Papillary muscle (PM) evaluated by three-dimensional echocardiography (3DE)

Variable	Patients with HC (*n* = 24)	Controls (*n* = 31)	*p* value
ALPM			
Number	1.7 ± 0.5	1.5 ± 0.6	0.172
CSA (cm^2^)	1.92 ± 0.81	1.15 ± 0.47	0.001
Bifid appearance	3 (13%)	2 (6%)	0.352
PPM			
Number	1.9 ± 0.4	2.1 ± 0.4	0.288
CSA (cm^2^)	1.46 ± 0.62	1.08 ± 0.37	0.031
Bifid appearance	3 (13%)	1 (3%)	0.144
Accessory PM	5 (21%)	3 (10%)	0.276

Data are presented as mean ± standard deviation or number (%)

*ALPM* anterolateral papillary muscle, *CSA* cross-sectional area, *HC* hypertrophic cardiomyopathy, *PM* papillary muscle, *PPM* posteromedial papillary muscle

In the group of 24 patients with HC, the median maximal LVOT gradient was 7 [IQR 4-30] mmHg. Six (25%) patients with HC demonsυtrated significant LVOT obstruction (≥ 30 mmHg). The PPM CSA was larger in patients with LVOT obstruction than in patients without (median 2.64 vs 1.16 cm^2^; *p* = 0.021). The ALPM CSA was not different between patients with and without LVOT obstruction (2.00 vs 1.92 cm^2^, *p* = 0.88).

## Discussion

This study demonstrates the utility of 3DE for the assessment of the geometric pattern of LV hypertrophy and PM morphology in patients with HC. Maximal LV hypertrophy was present at basal level in the infero- and anteroseptal segments, at midventricular level in the anteroseptal segments, and at apical level in the lateral segments. The hypertrophy in patients with HC followed a spiral pattern from the LV base to the apex. 3DE allowed evaluation of the PM morphology and hypertrophy and showed that patients with HC had significantly hypertrophied PMs as compared to healthy controls. Moreover, the amount of hypertrophy of the PPM was related to significant LVOT obstruction.

Previous studies have demonstrated that 3DE may be superior to 2DE and has a comparable accuracy to that of CMR for determining LV volumes, mass, and ejection fraction in various patient groups [10, 12–14]. Shimada and Shiota [15] performed a meta-analysis of the accuracy of 3DE for the measurement of LV mass, including 18 articles with 25 studies. The meta-analysis showed that a significant improvement of the accuracy of the 3DE technique has been achieved over time. The improved accuracy in most recent studies on 3DE is probably related to an improvement in the temporal and spatial resolution of the probe, and a more developed data analysis method using updated software. The meta-analysis included two studies on the use of 3DE for the assessment of LV mass in patients with HC [10, 16]. Oe et al. [16] studied the accuracy of 3DE in 21 patients with LV hypertrophy (17 with HC, and 4 patients with hypertensive heart disease) using CMR as a reference technique. LV mass was estimated accurately and easily by 3DE, whereas LV mass assessed by 2DE correlated less well with CMR. Bicudo et al. [10] studied 20 patients with HC who underwent 2DE, 3DE and CMR. In that study, 3DE had a better performance than 2DE for the evaluation of LV hypertrophy, volumes, ejection fraction, and mass when compared to CMR. The current study extends the findings from these previous studies and demonstrates the utility of 3DE in patients with HC for the assessment of the geometric pattern of LV hypertrophy and PM morphology and hypertrophy. Hence, 3DE allows both the assessment of global LV hypertrophy (as measured by LV mass) as well as local hypertrophy (as measured by LV segmental wall thickness and PM CSA).

In this study, the amount and location of LV hypertrophy varied among patients with HC. Generally, a longitudinal spiral trajectory of the hypertrophy was observed. This is in line with a recent study by Florian et al. [17], who studied the geometry of hypertrophy on CMR in 132 patients with HC. Using 3D analysis, the majority of patients exhibited a spiral pattern of hypertrophy. As in the present study, the magnitude of hypertrophy and rotation was variable. This spiral distribution of the hypertrophy may be caused by predominant involvement of the subendocardial layers of the myocardium. These findings are in agreement with necropsy studies in patients who died from HC, showing that the greatest cellular hypertrophy was in the layers closest to the cavity [18]. Likely, the distribution of hypertrophy is related to the stress distribution in the LV endocardium and subendocardium.

Approximately 30–60% of the patients with HC has a resting or provable LVOT obstruction, which may cause symptoms [19]. LVOT obstruction in patients with HC may be caused by several factors. Clearly, basal septal hypertrophy and systolic anterior movement of the mitral valve leaflet may cause LVOT obstruction. But also anatomical variations and hypertrophy of the PM may contribute [20]. Anatomical variations such as anterior displacement of the PM, the presence of a double bifid PM, anomalous insertion of the PM onto the anterior mitral valve leaflet, or fusion of the PM and the septum or LV free wall may have hemodynamic consequences.

Traditionally, 2DE has been used to assess the factors causing LVOT obstruction, but additional information may be obtained by 3DE and CMR. Our study demonstrates that the PPM CSA was larger in HC patients with LVOT obstruction than in those without. Previous studies have reported similar findings. Harrigan et al. [21] obtained CMR images in 201 patients with HC and 43 controls, in order to characterize PM morphology. PM mass index was significantly increased in patients with HC compared with controls, and PM hypertrophy was most severe in patients with LVOT obstruction. Furthermore, Kwon et al. [22] studied 56 patients with HC and 30 controls using CMR. The presence of PM abnormalities on CMR was correlated with resting LVOT gradients obtained by Doppler echocardiography. Patients with HC and abnormal PMs had significantly higher resting LVOT gradients, independent of septal LVWT. The identification of PM abnormalities may be relevant to understand the pathophysiology of LVOT obstruction and could have therapeutic consequences in patients with HC and significant LVOT obstruction who are considered for septal reduction surgery. Kwon et al. [23] have suggested that symptomatic patients with HC and significant LVOT obstruction with abnormal PM morphology may need surgical PM reorientation instead of or combined with standard surgical procedures. According to a large surgical series reported by Minakata et al. [6], patients with HC associated with anomalous PMs or chordae, including accessory PMs, can be successfully treated by surgical relief of morphological anomalies and an extended septal myectomy without mitral valve replacement. Finally, the utility of 3DE was also shown for the measurement of mitral leaflet surface area and subvalvular geometry in patients with HC. Kim et al. [24] studied 47 patients with HC and 32 controls using 3DE, and demonstrated that abnormal PM-mitral valve geometry assessed by 3DE may provide reasonable new targets for individualized surgical intervention.

This study has several limitations. First, the study population was relatively small, which may have affected the results. Second, only patients with sufficient image quality were included in the study. Third, the study was conducted in a referral center for patients with HC and may have therefore caused a bias. Fourth, the patient group included significantly more men than the control group. Finally, research is needed to integrate the findings from 3DE imaging in clinical decision making in order to improve outcome of patients with HC.

In conclusion, 3DE allows the assessment of LV geometry and PM abnormalities in patients with HC. 3DE demonstrated that the maximum hypertrophy was variable and generally located in a spiral from the LV base to the apex. A better visualization of these structures may improve the understanding of the pathophysiology and may influence the surgical treatment of left ventricular outflow tract obstruction in these patients.
